# Urban-rural inequality regarding drug prescriptions in primary care facilities – a pre-post comparison of the National Essential Medicines Scheme of China

**DOI:** 10.1186/s12939-015-0186-7

**Published:** 2015-07-30

**Authors:** Qiang Yao, Chaojie Liu, J. Adamm Ferrier, Zhiyong Liu, Ju Sun

**Affiliations:** School of Political Science and Public Administration, Wuhan University, Wuhan, Hubei 430072 China; School of Psychology and Public Health, La Trobe University, Melbourne, VIC 3086 Australia; School of Medicine and Health Management, Tongji Medical College of Huazhong University of Science and Technology, Wuhan, Hubei 430030 China

**Keywords:** Primary health care, Physician’s practice patterns, Essential medicines, Inequalities, China

## Abstract

**Objective:**

To assess the impact of the National Essential Medicines Scheme (NEMS) with respect to urban-rural inequalities regarding drug prescriptions in primary care facilities.

**Methods:**

A stratified two-stage random sampling strategy was used to sample 23,040 prescriptions from 192 primary care facilities from 2009 to 2010. Difference-in-Difference (DID) analyses were performed to test the association between NEMS and urban-rural gaps in prescription patterns. Between-Group Variance and Theil Index were calculated to measure urban-rural absolute and relative disparities in drug prescriptions.

**Results:**

The use of the Essential Medicines List (EML) achieved a compliance rate of up to 90 % in both urban and rural facilities. An overall reduction of average prescription cost improved economic access to drugs for patients in both areas. However, we observed an increased urban-rural disparity in average expenditure per prescription. The rate of antibiotics and glucocorticoids prescription remained high, despite a reduced disparity between urban and rural facilities. The average incidence of antibiotic prescription increased slightly in urban facilities (62 to 63 %) and reduced in rural facilities (67 % to 66 %). The urban-rural disparity in the use of parenteral administration (injections and infusions) increased, albeit at a high level in both areas (44 %-52 %).

**Conclusion:**

NEMS interventions are effective in reducing the overall average prescription costs. Despite the increased use of the EML, indicator performances with respect to rational drug prescribing and use remain poor and exceed the WHO/INRUD recommended cutoff values and worldwide benchmarks. There is an increased gap between urban and rural areas in the use of parenteral administration and expenditure per prescription.

## Introduction

The World Health Organization (WHO) defines the rational use of drugs as “patients receiving medications appropriate to their clinical needs, in doses that meet their own individual requirements, for an adequate period of time, and at the lowest cost to them and their community” [1, 2]. Rational use suggests that drugs should only be used in relation to the right diagnosis and right treatment: administered in appropriate strength, dosage, frequency, duration, and route of administration that is most convenient to the patient, with the best possible outcome and the least possible risk of adverse outcomes [1, 3]. Rational use is predicated upon a reliable diagnosis based on the correct identification of a patient’s presenting signs and symptoms; consideration of potential management options (both drug and non-drug related); determination of appropriate drugs, dosage and duration; prescription; administration; and monitoring of effect with adjustment of treatment options as and when required [4].

Rational drug prescribing contributes to global reductions in population morbidity and mortality with consequential medical, social and economic benefits [5]. Unfortunately, the irrational use of drugs is endemic, especially in middle and low income countries [6, 7]. Common aspects of irrational drug use include misuse, overprescribing or poly-pharmacy (prescribing more species of drugs than strictly necessary), inadequate dosage, and inadequate duration of drug therapy [1, 3]. In 2010, the WHO reported that, more often than not, drugs are prescribed inappropriately, compounded by 50 % of all patients using their medicines incorrectly [8]. The misuse and overuse of antibiotics is a serious concern in China [9–11]. The frequency of antibiotic prescriptions in China is twice that of the indicator developed by the WHO. Patients in China are three times more likely to have prescriptions requiring administration by infusion and injections than those from similar countries [12].

There is a wide disparity in the rational use of drugs between high-income and low- and middle-income countries. The overuse of parenteral administration is particularly prevalent in low-income countries, alarmingly so when many cannot guarantee safety of this route of administration [9, 13]. The WHO reported that about 90 % of parenteral administrations are unnecessary as there are better routes available [12]. In China, similar gaps between urban (richer) and rural (poorer) areas exist. Dong, Yan, and Wang found that the overuse of antibiotics and parenteral administration (i.e. infusions and injections) in preference to the oral route are the most prominent manifestations of irrational drug prescription practices in rural areas of Western China [14]. Chen et al found that antibiotics prescribed without any indication of infection for adult patients during routine outpatient consultations in Shandong and Gansu provinces ranged from 34 to 77 %, and parenteral administration of antibiotics in those patients ranged from 22 to 61 % [15].

Many countries have developed and implemented strategies or policies to improve the rational drug use [16]. China developed a comprehensive National Essential Medicines Scheme (NEMS) in 2009, with an aim to control growth of drug expenditure and improve quality use of medicines [17]. The 2009 NEMS contains a list of 317 drugs that must be accessible by primary care providers for the benefit of consumers. In acknowledgement of the importance of this initiative it was nominated as one of the five priorities in healthcare reform strategy (2009–11) [18, 19]. The Central Government State Council established a national target for 30 % coverage of NEMS within public primary care institutions in 2009, aiming towards 100 % coverage by the end of 2011 [7]. By 2011, somewhat ahead of the target deadline, the national EML and province-based centralized-procurement systems for primary healthcare institutions had been established and were in use all over China. All public primary care institutions can only provide essential medicines listed in EML and all drugs must be directly purchased from accredited suppliers via centralized procurement arrangements.

Despite significant efforts in implementing these strategies, empirical experience to date suggests doubt regarding the effectiveness of the NEMS for achieving its policy goals. A three year study of the use of antibiotics, hormones, and intravenous parenteral administration in outpatient services and primary care institutions in 83 counties/cities showed variable and small changes in the institutions where NEMS had been adopted, compared with those that had not adopted such schemes [20]. Yang et al found that while the NEMS interventions in Hubei province reduced the cost of medicines, the overuse of antibiotics and parenteral administration remained at high levels [7]. Many contextual factors are believed to contribute to the failure to fully achieve the NEMS expectations [10, 12, 14, 15, 21–24].

This study aims to examine the urban-rural inequalities regarding drug prescriptions in primary care facilities and the impact of the NEMS on such inequalities. Understanding the potential effect of NEMS on possible inequalities in drug prescription practices between urban and rural primary care facilities is important to inform and prioritize policy interventions, because changes in use of medicines may occur more markedly in one particular subgroup of population than in others. Overall "improved" averages may also accompany wider disparities between regions - some regions may even be worse off.

## Methods

### Setting

This study was conducted in A province. A province is located in the middle-eastern of China, with a population of 56,493,891 (in 2010). The total gross domestic product (GDP) of the province was CNY¥ 1235.9 billion (US$ 181.8 billion) in 2010.

Primary care facilities were the principal sources of information for this study, which focused on urban community health centers (CHCs) and rural township health centers (THCs). The Chinese health system is characterized by a three tier healthcare delivery system. The urban three tier system comprises CHCs, district hospitals and municipal/regional hospitals, while the rural three-tier system comprises village clinics, THCs, and county hospitals [19, 25]. THCs are the crux of the rural service network, providing primary care services, medical care services and public health services, in addition to technical support and training to health workers of village clinics [26]. While CHCs are the urban counterpart of THCs, they occupy a foundational status within the urban three-tier network, although some CHCs may run outreach stations within communities, which resemble rural village clinics.

### Sampling

Data were collected in 2009 and 2010, samples preceding and following NEMS implementation. We considered CHCs and THCs in the 32 counties/districts where the NEMS was introduced on 1 January 2010 eligible for potential inclusion in the study.

The sample size was determined in accordance with the guideline of the WHO “*How to investigate drug use in health facilities: selected drug use indicators”* [27]. To assess the impact of interventions, at least 10 facilities (or 20 for a more reliable study) per group and 30 prescribing encounters per facility were recommended. Based on 80 % power to detect a significant difference of *p* = 0.05, a moderate effect size of 0.02 (2 percentage points change in prescription rate), 3520 prescriptions per group pre- or post-interventions were required [28]. The sample size of this study achieved a minimum of 3760 (for urban facilities).

A stratified two-stage random sampling strategy was used in the study. In the first stage, four urban districts and nine rural counties were randomly selected. All public primary care facilities (n = 192) in the selected districts/counties were included in the study (63 from urban districts, 129 from rural counties). In the second stage, we used a systematic random sampling method to collect 120 outpatient prescriptions at each health center. These included 20 prescriptions randomly selected on the 2th March, 6th July and 2th November 2009 respectively (prior to the introduction of NEMS), and 20 prescriptions randomly selected on the 1st March, 5th July, and 1st November 2010 respectively (after NEMS implementation on 1st January 2010) for each health center. The sampling of prescriptions was extended to the following day if the sample size did not reach 20. We specifically excluded prescriptions arising from consultations in the departments of emergency, communicable diseases and surgery, and those containing traditional Chinese medicine decoctions.

Data from 23,040 collected prescriptions were transferred into an electronic data spreadsheet: of these some 67 sampled prescriptions contained conflicting information or lacked key information and were therefore excluded. This resulted in a final sample of 22,973 prescriptions (7519 urban and 15,454 rural) for analysis.

### Data analysis

The WHO and International Network for Rational Use of Drugs (WHO/INRUD) developed a list of indicators [27, 29] that are widely used for assessing irrational or inappropriate prescribing [12]. We adapted four of the five prescribing indicators in this study [27]:

1. average number of drug species per prescription;
2. percentage of prescriptions requiring antibiotics;
3. percentage of prescriptions requiring injections; and
4. percentage of drugs prescribed from the EML.

The WHO/INRUD indicator regarding *prescription of generic drugs* was discarded because almost all medicines prescribed by the primary care facilities in China are generic medicines.

In response to overwhelming concerns from local health officials, we added two additional indicators:

5. percentage of prescription requiring glucocorticoids; and
6. average expenditure per prescription.

For this study, a ‘glucocorticoid’ was defined as a systemic administration of a glucocorticoid (oral or via parenteral route) and specifically excluded local or topical applications [19].

Outcomes in relation to the above indicators were compared on an annual comparative basis (before and after introduction of the NEMS) and between urban and rural facilities. Statistical significances of the comparative results were examined using Chi-Square tests for the categorical indicators (frequencies) and student t-tests or Analysis of Variance (ANOVA) for the continuous measurements (quantity and drug expenditure).

We adopted the difference-in-difference (DID) method to assess the impact of the NEMS on urban-rural inequalities regarding the prescription indicators, controlling for the characteristics of facilities (servicing radius, population and density, number and composition of staff, income of staff and organizations) and community profiles (age, sex, marriage, and income).

We used a logistic regression method (eq 1) for categorical indicators (such as indicator 1, 2, 3, and 4) and least squares regression model (eq 2) for continuous indicators (such as indicator 5 and 6).

1 $$ In\left(\frac{{P_i}_j}{1-{P_i}_j}\right)={\beta}_0+{\beta}_1{T_i}_j+{\beta}_2{A_i}_j+{\beta}_3\left({T_i}_j*{A_i}_j\right)+{\displaystyle \sum_1^P{\beta}_p{x}_P{{}_i}_j}{\displaystyle \sum_1^o{\beta}_0{x}_o{{}_i}_j} $$

2 $$ {y_i}_j={\beta}_0+{\beta}_1{T_i}_j+{\beta}_2{A_i}_j+{\beta}_3\left({T_i}_j*{A_i}_j\right)+{\displaystyle \sum_1^P{\beta}_p{x}_P{{}_i}_j}{\displaystyle \sum_1^o{\beta}_0{x}_o{{}_i}_j} $$

Where, *i* =1 . . . *i* prescription, *j* =1. . . *j* organization; *p*_*ij*_ represents the probability of the occurrence of a prescribing behavior (i.e., 1 yes or 0 no); T_*ij*_ indicates when the prescription was made (0 pre-NEMS or 1 post-NEMS); A_ij_ represents the setting in which the prescription was made (0 urban or 1 rural); p =1. . . p community covariate, o =1. . .o organization covariate; x_oij_ and x_pij_ are control variables. Coefficient β3 represents the DID coefficient and reflects the effect of the NEMS on urban-rural differences in the prescribing indicators.

Between-Group Variance (BGV) [30, 31] and Theil Index (TI) [32, 33] were calculated to measure urban-rural absolute inequality and relative inequality, respectively. A higher value of BGV and TI indicates a higher level of inequality [34]. The formulas are as follows:

3 $$ BGV={\displaystyle \sum_{j=1}^J}{p}_j{\left({y}_j-\mu \right)}^2 $$

where *p*_j_ represents group j’s population size, *y*_j_ represents group *j*’s average status, and *u* represents the average value all the population [35].

4 $$ TI={\displaystyle \sum_{j=1}^J}{p}_j{r}_j \ln {r}_j $$

where *p*_*j*_ represents the proportion of population of group *j* in total and *r*_*j*_ represents the ratio of a condition in group j relative to the total [35].

The analyses were performed using Stata software version 13.0 [36] and Health Disparities Calculator version 1.2.4 [37].

This study was approved by the Ethical Review Committee of Tongji Medical College, Huazhong University of Science and Technology (IORG No: IORG0003571).

## Results

### Characteristics of participating primary health centers

The magnitudes of client populations and geographic areas are often considered defining differences: a higher client population density was shown in urban facilities compared with their rural counterparts (*p* < 0.001). Urban primary care facilities in this study served an area ranging on average from 270 to 491 km^2^, whereas rural areas were far larger from 470 to 763 km^2^. Urban facilities also had more staff (*p* < 0.05) and doctors (*p* < 0.01), and higher levels of annual revenue (*p* < 0.05) and salaries for medical staff (*p* < 0.001) than rural facilities. Despite this, the average revenue generated from drug prescriptions was slightly higher in rural facilities (CNY¥ 1,149,600) compared to urban facilities (CNY¥ 1,122,600), but without statistical significance (*p* = 0.256).

Both urban and rural facilities had at least 166 species of drugs from the EML. Urban primary care facilities had a greater range of drugs both from within and in addition to the EML than their rural counterparts. National EML drugs contributed to a similar proportion (57 %-60 %) in the complete range of drugs available (Table 1).

**Table 1:** Characteristics of participating primary care facilities in urban and rural areas

Indicator	Urban (95 % CI)			Rural (95 % CI)			*p* value
Client population (thousand)	46.97	(42.66,	51.29)	43.49	(39.00,	47.99)	**0.014**
Serving radius (km)	10.88	(9.27,	12.50)	13.91	(12.23,	15.58)	**0.013**
Population density (people/km^2^)	1036.15	(474.33,	1597.97)	248.01	(203.08,	292.94)	**0.000**
Number of medical staff	43.38	(38.53,	48.24)	41.30	(36.46,	46.15)	**0.013**
Number of doctor	17.69	(15.18,	20.20)	13.83	(12.32,	15.34)	**0.001**
Monthly income of medical staff (yuan)	2161.68	(2041.10,	2282.26)	1812.13	(1737.93,	1886.33)	**0.000**
Annual income of facility (thousand yuan)	3729.41	(3235.44,	4223.38)	3185.05	(2864.58,	3505.52)	**0.020**
Drug income of facility (thousand yuan)	1122.60	(896.87,	1348.34)	1149.62	(1009.00,	1290.24)	0.256
Variety of drugs in stock	324.13	(292.18,	356.08)	294.30	(276.33,	312.26)	0.753
Variety of drugs from national EMLs	183.98	(166.22,	201.74)	175.98	(166.00,	185.96)	0.366

Bold indicates statistical significance (*p* < 0.05)

Bold indicates statistical significance (p < 0.05)

### Urban-rural inequalities in prescribing indicators

Urban-rural inequalities existed prior to the NEMS (Table 2): primary care providers in rural facilities prescribed more varieties of drugs per prescription (p < 0.001) and were more likely to prescribe antibiotics (*p* < 0.01) and drugs from the EML (p < 0.001). But no significant urban-rural differences in glucocorticoids prescription, parenteral administration of drugs, and average cost per prescription were found (*p* > 0.05). The average prescription price to the consumer was about CNY¥ 42 (about US$6 in 2009) for both urban and rural patients.

**Table 2:** Prescribing indicators in primary care facilities in A province of China - pre and post NEMS

Indicator	Urban (95 % CI)		Rural (95 % CI)		Urban-rural difference				NEMS effect	
	Pre	Post	Pre	Post	Adjusted beta	(95 % CI)		*p* value	Pre *p* value	Post *p* value
Average number of drugs per prescription	3.61	3.45	4.33	4.15	-0.079	(-0.230,	0.073)	0.311	**0.000**	**0.000**
Percentage of prescriptions with antibiotics prescribed (%)	61.63	62.51	67.26	66.05	-0.154	(-0.286,	-0.021)	**0.023**	**0.009**	0.152
Percentage of prescriptions with glucocorticoids prescribed (%)	19.97	19.37	23.99	22.1	-0.135	(-0.296,	0.026)	0.101	0.081	0.428
Percentage of prescriptions with injections prescribed (%)	44.95	44.04	51.07	52.35	0.006	(-0.127,	0.139)	0.329	0.237	**0.003**
Percentage of drugs prescribed from the essential drug list (%)	66.71	88.28	75.4	92.3	-0.298	(-0.572,	-0.025)	**0.032**	**0.000**	0.412
Average expenditure per prescription (yuan)	42.12	28.2	42.74	32.73	2.869	(0.450,	5.289)	**0.020**	0.856	**0.000**

Bold indicates statistical significance (*p* < 0.05)

The DID tests demonstrated significant effects of NEMS on urban-rural differences (*p* < 0.05) in the use of antibiotics and EML, and prescription costs (Table 2). The pre-existing urban-rural disparity in prescriptions containing antibiotics declined, due to a slight decrease in urban and a corresponding increase in rural facilities. The proportion of prescriptions from the EML reached a consistent high level of nearly 90 % in both urban and rural facilities. However, significant urban-rural gaps in prescription costs appeared after NEMS implementation. Despite a significant decline in expenditure associated with the prescriptions in both groups, rural facilities were more likely to order and prescribe more expensive drugs (*p* < 0.001) compared with their urban counterparts.

The emerging urban-rural difference in the use of parenteral administration of drugs (*p* < 0.01) post NEMS was found to be associated with controlling/confounding factors, not the NEMS itself (*p* = 0.329).

### Absolute and relative urban-rural inequalities

Mixed trends of area-based absolute inequality (Table 3) and relative inequality (Table 4) in drug prescription practices were found. Urban-rural absolute and relative disparities in the use of antibiotics (*p* < 0.05) and prescriptions from the EML (*p* < 0.05) decreased. Progress was also demonstrated in eliminating relative inequality in the proportion of prescriptions requiring glucocorticoids (*p* < 0.01); however, the absolute and relative inequality in prescription cost increased (*p* < 0.001). Disparities in the average number of drugs per prescription remained constant (*p >* 0.1).

**Table 3:** Area-based Between-Group Variance (BGV) for drug prescription indicators - pre and post NEMS

Indicator	Pre (95 % CI)			Post (95 % CI)			*p* value
Average number of drugs per prescription	0.113	(0.083,	0.143)	0.106	(0.079,	0.133)	0.746
Percentage of prescriptions requiring antibiotics (%)	6.979	(2.300,	11.658)	2.764	(-0.212,	5.739)	**0.024**
Percentage of prescriptions requiring glucocorticoids (%)	3.558	(0.709,	6.408)	1.638	(-0.283,	3.559)	0.206
Percentage of prescriptions requiring injections (%)	8.236	(2.972,	13.500)	15.163	(8.053,	22.273)	0.668
Percentage of drugs prescribed from the essential drug list (%)	16.656	(10.502,	22.811)	3.551	(1.083,	6.018)	**0.037**
Average expenditure per prescription	0.085	(-0.651,	0.821)	4.52	(2.182,	6.859)	**0.000**

Bold indicates statistical significance (*p* < 0.05)

**Table 4:** Area-based Theil Index (×10000) for drug prescription indicators - pre and post NEMS

Indicator	Pre (95 % CI)			Post (95 % CI)			*P* value
Average number of drugs per prescription	34.517	(25.355,	43.678)	35.208	(26.052,	44.365)	0.171
Percentage of prescriptions requiring antibiotics	8.239	(2.714,	13.765)	3.303	(-0.204,	6.810)	**0.005**
Percentage of prescriptions requiring glucocorticoids	35.392	(7.047,	63.736)	18.499	(-2.897,	39.894)	**0.005**
Percentage of prescriptions requiring injections	17.367	(6.236,	28.499)	31.421	(16.536,	46.306)	0.246
Percentage of drugs prescribed from the essential drug list	16.052	(10.148,	21.957)	2.156	(0.675,	3.636)	**0.040**
Average expenditure per prescription	0.235	(-1.160,	1.631)	23.564	(11.289,	35.840)	**0.001**

Bold indicates statistical significance (*p* < 0.05)

Relative measures are without units and could be used to compare the inequality of indicators with different units [34]. As shown in Table 4, it is clear that the relative inequality of average number of drug per prescription is at a much higher level than that of other indicators both in 2009 and 2010. Meanwhile, increased and higher levels of relative inequality were also found in 2010 regarding prescriptions requiring injections and average expenditure per prescription.

**Fig. 1:**
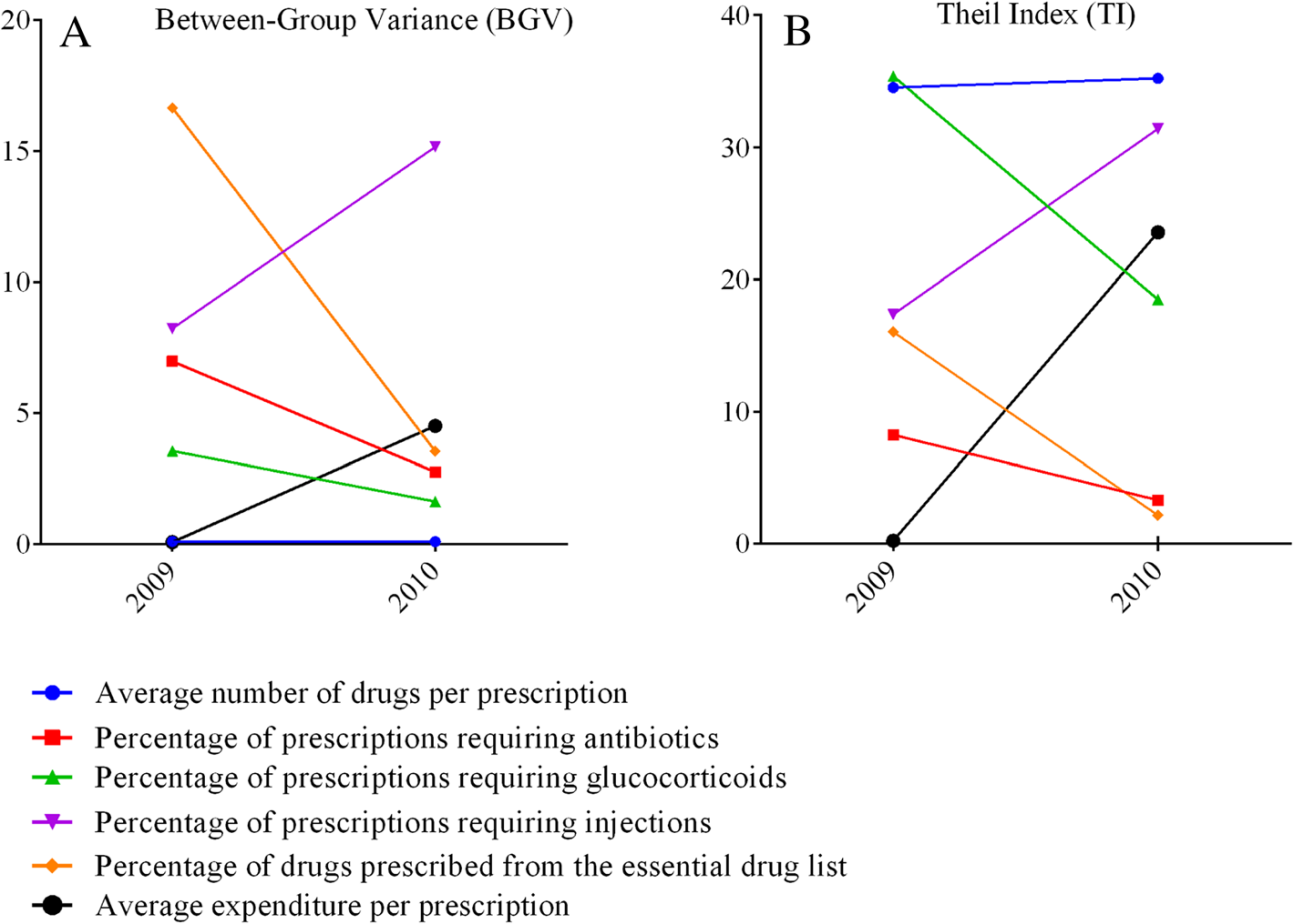
Area-based inequality trends. Changes of area-based inequality regarding drug prescription indicators, 2009-2010

Absolute and relative inequality measures are usually reported together for relative indicators could be used to make comparisons between indicators with different units and absolute indicators reflect the concrete difference value between subgroups [34]. Fig. 1 demonstrates that increasing urban-rural absolute and relative inequalities is evident for parenteral administration and prescription expenditure. A consistently high level of urban-rural relative inequality was observed for the “number of drugs per prescription”.

**Fig. 2:**
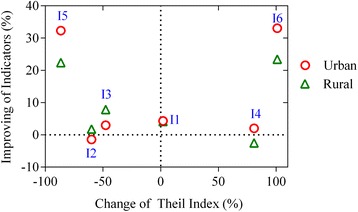
Four-quadrant view: improving of indicators vs urban-rural inequality. Four-quadrant view of drug prescription indicators: improving of indicators versus urban-rural inequality, 2009-2010. Group (a): I5 - Percentage of drugs prescribed from the essential drug list, shows improvement in both average level and equality; Group (b): I2 - Percentage of prescriptions requiring antibiotics, I3 - Percentage of prescriptions requiring glucocorticoids: these -indicators show improved urbanrural equality but with limited changes in average levels Group (c): I6 - Average expenditure per prescription, shows improvement in average level but with enlarged inequality; Group (d): I4 - Percentage of prescriptions requiring injections, shows poor performance in both average level and equality; Group (e): I1 - Average number of drugs per prescription, shows little changes in both average level and equality

By using “average improving of indicators” and “changes of relative inequality”, all indicators were placed into a four-quadrant view (Fig. 2). The upper left group (a) indicates improvement in both average levels and equalities of indicators. The proportion of drugs prescribed from the EML fell into this group. The lower left group (b) reflects improved urban-rural equality but with limited changes in average levels. Attention should be paid to the improvement of the levels of those indicators, such as the proportion of prescriptions requiring antibiotics or glucocorticoids. The upper right group (c) indicates improvement in average levels but with enlarged inequalities of indicators. Average expenditure per prescription fell into this group. The lower right group (d) represents the worst performed indicators in both average levels and equalities. Prescriptions requiring injections fell into this group. The final group (e) stands in the middle, indicating limited changes in both average levels and inequalities. The average number of drugs per prescription fell into this group.

## Discussion

This study assesses the impact of the NEMS on urban-rural inequalities regarding drug prescriptions in primary care facilities, and we found a mixed picture.

The most prominent achievement is the compliance with the EML. Both urban and rural facilities have increased their use of EML, reaching a compliance rate of about 90 %. The former urban-rural disparity for this indicator is no longer present. Our results indicate that proportion of drugs prescribed from the EML increased from 66.71 to 88.28 % in urban areas and from 75.40 to 92.30 % in rural areas, a performance outcome higher than Western Pacific countries (75.5 %), South East Asia (77.0 %), and lower-middle income countries (81.7 %). These results agree well with the findings of Yang LP, et al [7]. We suspect limited choices and availability of medicines contributes to this effect: NEMS specifies that pharmaceutical supply in primary care facilities are restricted to only those drugs listed in the EML.

Another important achievement is the reduction of expenditure per prescription, which may be a result of a proportional increase of EML drugs and reduction of drug stocks; this finding is consistent with earlier findings [7, 38]. The four elements of NEMS may contribute to the overall reduction of prescription expenditure, including EML, province-based centralized procurement initiatives, zero-mark-up policy, and reimbursement arrangements for EML drugs. All drugs on the NEMS list are subject to the zero-mark-up policy, and EML drugs usually have a higher reimbursement rates.

Overall, NEMS improved the economic access of drugs in both rural and urban areas in the province investigated; however, it appears that benefits are not equally distributed. Urban residents enjoy a greater cost reduction than their rural counterparts. One possible reason for this could be the differences in medical insurance between urban and rural areas. The reimbursement ratio of Urban Employee Basic Medical Insurance is higher than that of New Cooperative Medical Scheme in rural areas. Moreover, drug supply logistics are challenging in remote and rural areas due to higher distribution costs. As a result, urban primary care facilities are able to pass on a higher level of benefits onto their patients than their rural counterparts.

The urban-rural gap in the use of antibiotics and glucocorticoids closed; however, antibiotic use in urban areas showed no reduction. It is a challenge to estimate the seriousness of irrational use of drugs from the prescribing indicators without proper risk-adjustment: we note the role of the WHO in establishing benchmarks. The WHO/INRUD suggests an indicator of optimal incidence of antibiotic prescriptions at less than 30 % of all prescriptions [14]. Currently, the percentage of prescriptions containing antibiotics (62.51 % urban, 66.05 % rural) exceeds these benchmarks (Table 5) [14, 39].

**Table 5:** Prescribing indicators in comparison with other countries and WHO/INRUD standard

Region	Average number of drugs per prescription	Percentage of prescriptions requiring antibiotics (%)	Percentage of prescriptions requiring injections (%)
Western Pacific	2.8	50.80	27.10
South East Asia	2.4	47.90	9.70
Lower-middle income countries	2.8	50.00	21.70
A province China (urban)	3.5	62.51	44.04
A province China (rural)	4.1	66.05	52.35
WHO/INRUD recommendation	1.6 – 1.8	<30	<10

Source of data: Holloway K, Ivanovska V, Wagner A, Vialle‐Valentin C, Ross‐Degnan D. Have we improved use of medicines in developing and transitional countries and do we know how to? Two decades of evidence. Tropical Medicine & International Health. 2013;18(6):656-64

Unexpectedly, the urban-rural gap in the use of injections increased with little decline in urban area and even worse in rural area. Meanwhile, the overall incidence of parenteral administration (44.04 % urban, 52.35 % rural) remains much higher than the WHO optimal value (<10 %) [14]. This finding is similar to the previous studies of Yang LP et al [7] and Li Y et al [38]. This study suggests that the NEMS is not associated with the increased urban-rural disparity in unnecessary parenteral administration. Indeed, both doctors and patients may play a role for the overuse of drug administration via injections or infusions. Doctors in urban areas usually have relatively higher levels of education and medical training, which appears to be associated with lower levels of non-oral administration than others [21]. In China, most patients, especially in rural areas, believe that infusions and injections are more efficacious than oral medicines, which in turn exerts an ethical dilemma for doctors who might otherwise recommend the oral route [24].

Finally, the urban-rural disparity in the average number of drugs per prescription remains unchanged. The average number of drugs prescribed, especially in rural areas, remains high compared to Western Pacific (2.8), South East Asia (2.4), and lower-middle income countries (2.8), and far from the WHO/INRUD recommended value: 1.6-2.8 [39, 40].

## Conclusion

This study shows that the NEMS interventions reduced overall average patient expenditure and increased the use of EML drugs by prescribers. But the cost benefits are not equally distributed: urban patients enjoy a greater cost reduction than their rural counterparts. Improvements in other indicators are absent. Prescription rates associated with antibiotics and glucocorticoids remain high despite reductions in urban-rural disparities. Both urban and rural practices exceed WHO/INRUD recommended cutoff values and worldwide benchmarks. The rate of parenteral drug administration in both urban and rural areas remains high compared with the WHO benchmarks.

The effectiveness of the NEMS is dependent upon multi-faceted strategies including education, managerial and financial interventions, and regulatory policies. Educational strategies targeted towards prescribers include standard treatment guidelines, flow charts, newsletters, bulletins and printed information (such as leaflets) [39, 41–43]. Managerial strategies include EML, kit system distribution, pre-printed order forms, stock control, course-of-therapy packaging, and effective package labeling [44, 45]. Regulatory strategies involve, for example, banning unsafe drugs, and limiting the import of drugs on the domestic market [44, 45]. However, the effectiveness of current financial incentives and interventions remains uncertain and problematic [44]. Unfortunately, Chinese township health centers lack a sustainable and developmental financial compensation mechanism for NEMS. As a result, many highly qualified health workers are leaving their institutions and some are departing medical practice. It would seem reasonable that a long-term compensation mechanism must be considered [7].

### Limitations

This study attempts to examine the impact of the NEMS on inequalities in quality use of medicines between urban and rural primary care facilities in China. The characteristics of health organizations and community profiles were controlled for potential confounding effects; however, due to limited availability of data we were unable to control the potential confounding effect of the characteristics of prescribers, such as their age, education and working experiences. This study was undertaken in one region of China: further studies are needed to assess inequality among different regions.
